# Human Telephone vs Text Message Counseling and Physical Activity Among Midlife and Older Adults: A Randomized Clinical Trial

**DOI:** 10.1001/jamanetworkopen.2025.28858

**Published:** 2025-09-04

**Authors:** Abby C. King, Maria Ines Campero, Patricia Rodriguez Espinosa, Dulce Garcia, Cecilia Corral, Cynthia Castro Sweet, Lan Xiao, Michael F. Royer, Astrid Zamora, Ana L. Cortes, Monica Done, Jorge A. Banda

**Affiliations:** 1 Department of Epidemiology & Population Health, Stanford University School of Medicine, Stanford, California; 2 Department of Medicine, Stanford Prevention Research Center, Stanford University School of Medicine, Stanford, California; 3 CareMessage, San Francisco, California; 4 Now with Hinge Health, San Francisco, California; 5 Now with Department of Pediatrics, Stanford University School of Medicine, Stanford, California; 6 Now with Department of Psychology, University of Nevada, Las Vegas; 7 College of Health and Human Sciences, Purdue University, West Lafayette, Indiana

## Abstract

**Importance:**

While walking and similar physical activities provide numerous health benefits, particularly for aging inactive adults, many interventions are not accessible or relevant for all populations. Expanding the range of effective programs represents a critical goal.

**Objective:**

To test whether counseling delivered by a customized, computer-driven short message service (SMS) system is comparable to standard human telephone counseling for increasing 12-month walking among inactive Hispanic or Latino/a adults.

**Design, Setting, and Participants:**

This parallel-group randomized equivalence clinical trial enrolled adults from November 2015 to September 2017, with follow-up through October 2018. Data analysis was performed from January 2023 to December 2024. The evidence-derived equivalence margin was 30 minutes of walking per week. Participants were inactive adults ages 35 to 79 years of primarily Latin American descent from 5 northern California counties.

**Interventions:**

Participants were randomized to receive a customized version of the evidence-supported Active Choices physical activity telephone advising program, delivered through trained human advisors or an interactive computerized SMS (text-messaging) system for 12 months.

**Main Outcomes and Measures:**

The primary outcome was change in total volume of walking minutes per week at the end of the 12-month interventions using validated interview assessment corroborated with accelerometry. Intention-to-treat principles were used.

**Results:**

Among 280 randomized participants (203 [72.5%] female; mean [SD] age, 51.2 (8.9) years), 139 were randomized to a human phone advisor and 141 were randomized to the SMS intervention. More than half of participants (145 participants [51.8%]) reported household income in the very low income range, and the mean (SD) body mass index was 33.1 (5.0). A total of 249 participants (88.9%) completed the study. At 12 months, mean (SD) walking increased 116.4 (2-sided 90% CI, 92.3 to 140.5) minutes per week (*P* < .001) for the human advisor cohort and 113.6 (2-sided 90% CI, 89.8 to 137.4) minutes per week (*P* < .001) for the SMS cohort (difference, 2.8 [90% CI, −23.8 to 29.4] minutes per week; *P* = .86). This finding supports equivalence. Benefits occurred in both groups for weight maintenance, sedentary behavior, and well-being outcomes, as well as additional physical activity types.

**Conclusions and Relevance:**

In this study of SMS vs human phone advising, a customizable SMS system produced significant 12-month walking increases for aging Latino/a adults comparable to the significant improvements attained by participants in the human advisors group. These results provide support for such mobile health platforms, which can expand program choices for broader segments of the population.

**Trial Registration:**

ClinicalTrials.gov Identifier: NCT02385591

## Key Points

**Question:**

Can a customized computer-driven 12-month short message service (SMS) counseling platform increase weekly walking minutes among adults to levels comparable with those receiving telephone counseling from a trained human advisor?

**Findings:**

In this randomized equivalence clinical trial of 280 midlife and older adults who self-identified as Hispanic or Latino/a, the computer-based SMS program achieved significant 12-month increases in weekly walking that were comparable to those achieved by participants who received telephone counseling from human advisors. Both programs helped promote weight maintenance and well-being outcomes.

**Meaning:**

The findings showing meaningful 12-month physical activity increases in the SMS and telephone programs expand the technology-enabled, customizable light touch program choices that can be offered to adults.

## Teaser

This randomized equivalence clinical trial examines whether counseling delivered via automated short message service vs telephone calls with a human advisor increases physical activity in Hispanic or Latino/a adults.

## Introduction

Few health behaviors have as broad impacts on health as regular physical activity (PA), demonstrated as an independent risk factor associated with all-cause mortality, cardiovascular disease, and numerous other outcomes.^1,2^ While PA guidelines emphasize accessible activities, like walking,^3,4^ 80% of US adults are insufficiently active^5^—particularly older, lower-income, and Latino/a adults, often due to inaccessible, 1-size-fits-all programming.^1,6^

The explosion of digital health programs^7,8^ allows an unparalleled opportunity to reach more populations with customized evidence-based programs, freeing participants from travel requirements and increasing accessibility. Yet, most digital health programs are targeted primarily at well-educated, largely English-speaking individuals.^9^

Short message service (SMS; ie, text-messaging) remains an ubiquitous, lower-cost platform across virtually all US population segments.^10^ Prior SMS programs have reported encouraging PA results.^1,11,12,13^ However, few sufficiently use behavioral theory, are customized for different groups,^14^ test SMS alone, use rigorous active comparison groups,^13^ or evaluate effectiveness beyond 3 to 6 months.^1,11,12,13^ Therefore, the On The Move trial systematically tested the 12-month effectiveness of a customized phone-based PA advising program delivered via standard human phone advisors vs computer-driven SMS.^15,16,17^

## Methods

This randomized equivalence clinical trial was approved by the Stanford University institutional review board. All participants provided written informed consent in their preferred language (English or Spanish). Participants received a $20 gift card per assessment (3 assessments). Such modest remuneration minimally impacts PA change.^1^ This study is reported following the Consolidated Standards of Reporting Trials (CONSORT) reporting guideline. The trial protocol and statistical analysis plan are available in Supplement 1.

### Design

This single-blind, parallel group randomized equivalence trial^18^ (1:1 allocation ratio) compared 2 different communication modalities (SMS advising vs standard human phone advising) delivering an evidence-supported PA program to Latino/a adults.^17,19^ It was conducted by Stanford University in collaboration with CareMessage, a nonprofit organization delivering mobile health interventions for low-income populations.^20,21,22^ Participants were enrolled from November 13, 2015, to September 26, 2017, from 5 San Francisco Bay–area counties using customized mailings, English- and Spanish-language media, and community outreach.^23,24,25^ Follow-up ended October 2, 2018. Participants completed online or telephone-based screening to establish eligibility, which included ages 35 years and older; insufficiently active^26^ (ie, <125 minutes per week of moderate intensity activity over the previous 6 months^27^); able to participate safely in walking and other moderate forms of PA (based on the PA Readiness Questionnaire^28^); able to read and understand English or Spanish sufficiently to provide informed consent and participate in procedures; remaining in the area for the next 12 months; self-reported body mass index (BMI; calculated as weight in kilograms divided by height in meters squared) between 25 and 46; not pregnant, planning a pregnancy, or had a newborn in the past year; and self-identified as Latino/a or Hispanic. Individuals were ineligible if they answered yes to safety screening items.^29^ Following informed consent and baseline evaluation, individuals were stratified by sex to ensure a comparable distribution of males and females in each group and randomized by trained staff blinded to assessments using a computerized Efron procedure^30^ (trial protocol in Supplement 1) to PA study group (human telephone advisor or SMS advisor) constituting the primary equivalence comparison, or a smaller SMS nutrition group aimed at exploring secondary questions of interest related specifically to the SMS communication channel. Comparing the 2 PA groups was the primary outcome of this investigation. Randomization allocation was concealed from assessment staff via anonymized identification numbers to minimize selection and measurement bias.

### Interventions

The primary intervention focus was total walking volume, given its convenience and health benefits for aging inactive adults.^5,6,31,32^ A 2024 meta-analysis^33^ found overall PA volume, including walking, of any intensity to be more strongly associated with lower mortality risk than moderate-to-vigorous PA (MVPA) in midlife and older adults.^33^ The 2 PA interventions were based on the extensively tested Active Choices PA program, drawn from behavioral theory^34,35^ and demonstrated over 3 decades to significantly increase PA in various populations^36,37,38,39,40^ (trial protocol in Supplement 1). It is grounded in tested principles of progression across frequency, intensity, and duration of relevant PA behaviors for the target population.^15,16,41^ The human telephone advisor program followed standard Active Choices procedures,^19^ with the number of 12-month total advising sessions customized to each participant’s preferences, ranging from 10 to 24 sessions based on prior Active Choices interventions.^37,39^ The SMS PA program used a similar set of Active Choices strategies, adapted for an SMS platform, with number and timing of text messages customized to each participant’s preferences (trial protocol in Supplement 1). CareMessage^20,21,22^ collaborated on messaging and provided the SMS platform. Feasibility and acceptability of SMS content were confirmed in a prior pilot study with 30 adults from the target population.^19^ Based on the pilot, a reasonable text message range was between 2 and 5 texts per week.

Both programs allowed for tailoring of scheduling and contact frequency to accommodate participant needs. After each group received a 30-minute introductory PA counseling session, human PA advisors called their participants on a monthly to semimonthly basis, with contacts lasting approximately 15 minutes. SMS advisor participants received 2 to 5 texts per week across 12 months. Content in both groups included personalized goal-setting, advice, feedback, prompts to identify role models and family values around PA, and support for PA change. In both groups, participants were given a pedometer and calendar as additional motivational tools.

### Outcome Assessments

Participants completed assessments at Stanford Medical School and community centers in the participating counties. Trained assessment staff were blinded to participant study group and prior data.

The primary outcome, 12-month changes in total weekly walking minutes, was assessed using the validated, bilingual Community Healthy Activities Model Program for Seniors (CHAMPS) instrument (interview format).^27,41,42^ This self-report instrument was explicitly developed to reliably assess the specific PA targeted in interventions for aging adults and has demonstrated sensitivity to change in diverse populations, including Latino/a individuals and those with lower incomes or education.^37,38,39,41,43,44^ In contrast, device-based assessment instruments (accelerometers) capture general movement levels, which may be unrelated to the intervention,^41,45,46^ can be less sensitive to change in underactive populations^47^ and burdensome for some aging adults,^43^ and lack sufficient normative data among Latino/a individuals. CHAMPS is among the few PA instruments for aging adults correlated with both criterion standard doubly labeled water-measured PA energy expenditure and accelerometers^47^ (trial protocol in Supplement 1).

Secondary outcomes included CHAMPS-derived MVPA, total PA, and percentage of participants meeting national PA recommendations of at least 150 minutes per week of MVPA.^48^ The validated Actigraph accelerometer (model wGT3X; Ametris) passively collected overall daily movement amounts and intensity (although not PA types) at the assessment time points.^19,41^ The accelerometer protocol was derived from a large study of 860 older US adults.^45^ Participants were instructed to wear it on the hip for at least 8 hours per day during waking hours for 7 consecutive days per time point, commensurate with studies aimed at expanding data inclusivity, especially among understudied, time-constrained populations.^49,50^ Wear-time validity was assessed by applying the wear and nonwear time analysis and classification algorithms reported by Choi et al.^51^

Other secondary outcomes^19^ included sedentary behavior, assessed using a validated 1-week recall survey^52^; BMI from standard clinical assessment protocols for height and weight^42^; abdominal adiposity (waist circumference)^53^; resting blood pressure and heart rate, assessed using standard protocols^42^; behavioral and social support strategies promoting positive PA change^6^; and quality of life and well-being, measured using the World Health Organization Quality of Life questionnaire (brief version).^54,55^ Participant 12-month program satisfaction ratings were assessed using the Working Alliance Inventory 12-item bonding subscale (range, 0-7; higher score indicates greater satisfaction and bonding).^43,56^

Sociodemographic information was collected using standard questionnaires.^57^ Program safety and adverse events were tracked at each assessment using standardized protocols.^38,39^ To evaluate program use, the SMS advisor automatically recorded the number of texts sent, texts with failed delivery, and texts with interactive components that did or did not receive a response.^19^ Human advisors systematically recorded number of calls attempted and successfully completed and total call lengths.^36,37,38,39,57^

### Intervention Fidelity and Quality Assurance

To ensure intervention fidelity, human advisors completed structured checklists and logs following each contact, with weekly supervisor-led intervention meetings to discuss questions and challenges. Supervisors periodically reviewed advising session audio recordings collected in all sessions with participant consent and performed random check-ins with participants.^19^ Quality assurance activities in the SMS group included regular system monitoring and backup, tracking of participant text-related response rates indicating text receipt and review, and availability of a helpline for any problems (trial protocol in Supplement 1).

### Statistical Analysis

Data analysis was performed from January 7, 2023, to December 20, 2024. A sample size of 280 participants (approximately 140 per group) was identified to provide greater than 80% power to determine 12-month equivalence of the 2 groups assuming 15% study attrition rates.^19^ Based on the literature,^1^ a clinically meaningful difference between groups of 30 minutes of total walking minutes per week with an estimated within-group SD of 100 (Cohen *d* effect size, 0.30) was used to determine sample size. This margin was based on previous Active Choices studies showing significant differences of more than this between this intervention and non-PA/attention control groups^37,38,39,58^ and literature indicating that with increasing episodes of PA per week (with such episodes typically lasting about 30 minutes^26,37^), risk of all-cause mortality, cardiovascular disease, and other chronic disease outcomes decrease.^1,59^ Extreme values of the study outcomes were limited by winsorization to 3 SDs above the mean.^60^ Missing outcomes were imputed using fully conditional specification methods with appropriate regression models, with 10 imputations combined.^61^ RCS was implemented using SAS procedure PROC MI (statistical analysis plan in Supplement 1). All reported outcomes were based on intention-to-treat principles.

Baseline characteristics were summarized using descriptive statistics, including independent-sample *t* tests for continuous variables and χ^2^ tests for categorical variables. The primary equivalence hypothesis^18^ was tested using a mixed-effects linear regression model that incorporated 6- and 12-month data using SAS statistical software version 9.4. Change in total walking minutes per week over 12 months was the primary outcome, with adjusted coefficients accounting for baseline PA value and the sex stratification variable. (Age, evaluated in an initial model, was not significant.) Using 2-sided 90% CIs constructed for the between-group difference in the primary outcome,^18^ equivalence was deemed established if the adjusted estimate lay within the equivalence margins of ±30 minutes per week of total walking.^62,63^ For completeness, both intention-to-treat and complete-case analyses are reported. Similar analyses were conducted for secondary variables. Within-group pre-post *t* tests and between-group 12-month testing of specific secondary outcomes (eg, program satisfaction) were conducted for descriptive purposes. The significance threshold was α = .05 using 2-tailed tests. Corroboratory analyses were conducted^64^ with accelerometry databased on intention-to-treat principles and taking into account data skewness in this sedentary sample (eResults in Supplement 2).

## Results

**Figure 1.**
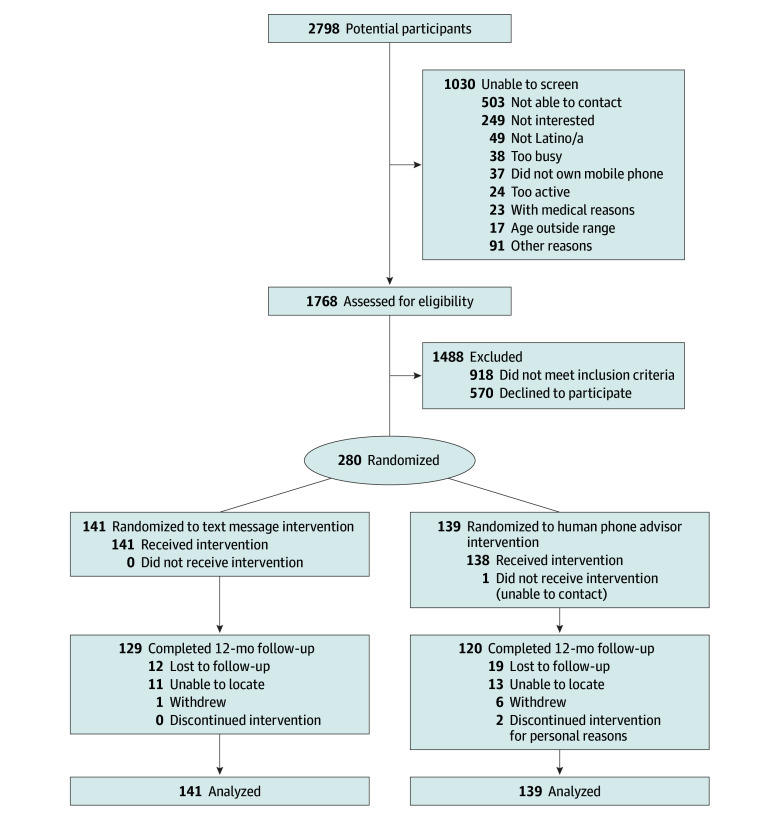
Participant Recruitment and Randomization Flowchart

A total of 280 participants (203 [72.5%] female; mean [SD] age, 51.2 (8.9) years) were enrolled, with 249 participants completing the 12-month data collection for the primary outcome, including 120 of 139 participants (86.3%) randomized to the human advisor and 129 of 141 participants (91.5%) randomized to the SMS advisor (Figure 1). Overall, 80 participants (28.6%) reported having 1 or more of the following: hypertension, past myocardial infarction, congestive heart failure, and/or vascular disease, and 38 participants (13.6%) reported type 2 diabetes. As per the study screening protocol, all participants reported being stable on medications for at least 3 months prior to enrollment and having fewer than 125 PA minutes per week. All participants self-identified as Latino/a, and 2 participants (0.7%) were African American, 3 participants (1.1%) were American Indian or Alaska Native, 217 participants (77.5%) were White, and 40 participants (14.3%) identified as more than 1 race (Table 1). A smaller proportion of completers, compared with noncompleters, were born in the US (37.8% vs 61.3%; *P* = .01), and completers had lower baseline total PA than noncompleters (mean [SD], 314.8 [237.0] vs 430.2 [321.0] minutes per week; *P* = .01).

**Table 1.** Baseline Characteristics

Characteristic	Participants, No. (%)		
	Overall (N = 280)	Human phone advisor (n = 139)	Interactive SMS advisor (n = 141)
Age, mean (SD), y	51.2 (8.9)	51.9 (9.1)	50.6 (8.7)
Sex			
Female	203 (72.5)	101 (72.7)	102 (72.3)
Male	77 (27.5)	38 (27.3)	39 (27.7)
Race			
African American	2 (0.7)	2 (1.4)	0
American Indian or Alaska Native	3 (1.1)	1 (0.7)	2 (1.4)
White	217 (77.5)	110 (79.1)	107 (75.9)
>1 Race^a^	40 (14.3)	20 (14.4)	20 (14.2)
Did not answer	18 (6.4)	6 (4.3)	12 (8.5)
Education			
<High school	26 (9.3)	12 (8.6)	14 (9.9)
High school or GED	58 (20.7)	28 (20.1)	30 (21.3)
Some college	85 (30.4)	46 (33.1)	39 (27.7)
College graduate	50 (17.9)	25 (18.0)	25 (17.7)
Post college	61(21.8)	28 (20.1)	33 (23.4)
Annual family income, $			
<25 000	24 (9.4)	9 (7.1)	15 (11.8)
25 000 to <35 000	13 (5.1)	8 (6.3)	5 (3.9)
35 000 to <50 000	36 (14.2)	20 (15.7)	16 (12.6)
50 000 to <75 000	58 (22.8)	29 (22.8)	29 (22.8)
>75 000	123 (48.4)	61 (48.0)	62 (48.8)
Missing	26	12	14
Marital status			
Never married	12 (4.5)	5 (3.8)	7 (5.1)
Married or living with a partner	209 (77.7)	106 (79.7)	103 (75.7)
Separated, divorced, or widowed	48 (17.8)	22 (16.5)	26 (19.1)
Missing	11	6	5
Employment status			
Employed	238 (96.7)	115 (97.5)	123 (96.1)
Retired	34 (12.1)	21 (15.1)	13 (9.2)
Other	8 (2.9)	3 (2.2)	5 (3.5)
Job type			
White collar	160 (58.0)	80 (58.0)	80 (58.0)
Blue collar	116 (42.0)	58 (42.0)	58 (42.0)
Missing	4	1	3
Born in US	113 (40.4)	61 (43.9)	52 (36.9)
Household size			
1	21 (7.5)	14 (10.1)	7 (5.0)
2	50 (17.9)	25 (18.1)	25 (17.7)
3	51 (18.3)	26 (18.8)	25 (17.7)
≥4	157 (56.3)	73 (52.9)	84 (59.6)
Missing	1	1	0
BMI, mean (SD)	33.1 (5.0)	33.0 (5.1)	33.3 (4.9)
Waist circumference, mean (SD), cm	106.4 (41.9)	105.9 (41.7)	106.9 (42.1)
Resting blood pressure, mean (SD), mm Hg			
Systolic	123.2 (15.3)	122.9 (14.5)	123.5 (16.0)
Diastolic	81.9 (9.6)	81.7 (8.4)	82.2 (10.6)
Resting heart rate, mean (SD)	71.0 (10.5)	71.6 (11.2)	70.5 (9.7)
Total time inactive, mean (SD), min/wk^b^	1952 (1263)	2058 (1339)	1848 (1179)
Time watching television/videos, mean (SD), min/wk^c^	601 (534)	630 (552)	572 (515)
CHAMPS-reported PA, mean (SD), min/wk			
Total walking	69.1 (95.9)	70.7 (99.1)	67.6 (93.0)
Moderate and vigorous PA	21.8 (41.9)	19.5 (38.8)	24.1 (44.7)
Total PA	327.5 (249.0)	329.8 (273.0)	325.3 (224.0)

Abbreviations: BMI, body mass index (calculated as weight in kilograms divided by height in meters squared); CHAMPS, Community Healthy Activities Model Program for Seniors; GED, general educational development; PA, physical activity; SMS, short message service.

^a^ More than 1 race selected included 6 individuals selecting American Indian or Alaskan Native and White; 1 individual, American Indian or Alaskan Native and Asian; 2 individuals; Black or African American and White; 2 individuals, Native Hawaiian or Other Pacific Islander and White; 20 individuals, did not state.

^b^ Inactive (sedentary) time total score range, 0 to 8760 minutes per week.

^c^ Inactive (sedentary) time item range, 0 to 3300 minutes per week.

Baseline participant characteristics were similar between groups (Table 1). Mean (SD) BMI was 33.1 (5.0) (range, 23.6-46.1). Approximately one-third of participants (84 participants [30.0%]) had a high school education or less, and more than half of participants (145 participants [51.8%]) reported a household income in the very low–income category for their area.

The human advisor group had a mean (SD) of 12.7 (6.7) sessions, representing 82.7% of intended sessions completed. The mean (SD) duration of each human advisor session was 14.8 (3.5) minutes, resulting in a mean (SD) of 186.1 (110.4) total minutes of advisor contact (ie, 3.1 hours). For the SMS group, a mean (SD) total of 183.1 (40.1) SMS messages were sent. Of the 48.6% of messages inviting a participant response, mean (SD) participant response rate was 94.1% (15.1%).

### Outcomes

#### Weekly Walking Minutes

**Figure 2.**
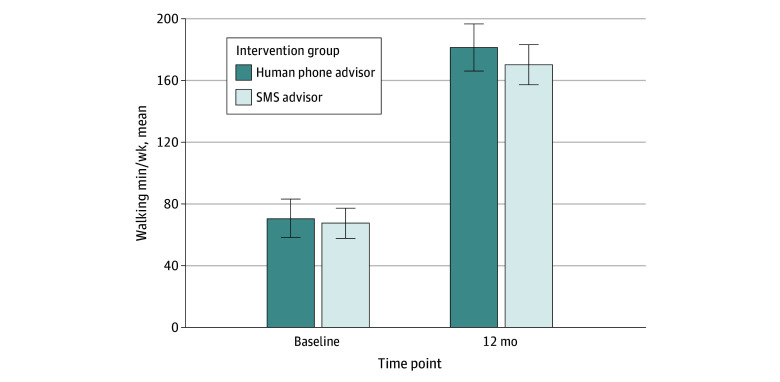
Mean Change in Total Walking Minutes Per Week by Intervention Group Raw means are shown, with vertical bars representing 2-sided 90% CIs. SMS indicates short message service.

Twelve-month change in total weekly walking minutes supported between-group equivalence, as the 2 end points of the 90% CIs lay between the population mean difference (Table 2 and Figure 2). Mean changes in weekly walking minutes were similar in the human advisor (116.4 [90% CI, 92.3 to 140.5] minutes per week) and SMS advisor groups (113.6 [90% CI, 89.8 to 137.4] minutes per week), with a between-group difference of 2.8 (90% CI, −23.8 to 29.4) minutes per week. Mean 12-month reported changes in MVPA minutes were also similar between groups (difference, −3.8 [90% CI, −26.7 to 29.4] minutes per week).

**Table 2.** Primary and Secondary Outcomes

Outcome	Intention-to-treat (with imputation)^a^				Complete-case analysis (without imputation)^b^					
	Mean (SE)		Difference (90% CI)	*P* value	Human phone advisor		Interactive SMS		Difference (90% CI)	*P* value
	Human phone advisor (n = 139)	Interactive SMS (n = 141)			No.	Mean (SE)	No.	Mean (SE)		
Total walking min/wk	116.4 (12.3)	113.6 (12.1)	2.8 (−23.8 to 29.4)	.86	120	118.6 (12.1)	129	112 (12.4)	6.6 (−20.4 to 33.6)	.69
Secondary outcomes										
Total MVPA, min/wk	103.3 (11.0)	107.1 (10.1)	−3.8 (−26.7 to 19.1)	.79	120	105.1 (10.5)	129	111.6 (10.0)	−6.4 (−29 to 16.2)	.64
Total PA, min/wk	122.1 (20.7)	117.8 (20.8)	4.3 (−40.7 to 49.3)	.88	120	117.4 (23.2)	129	120.1 (20.8)	−2.7 (−51.2 to 45.7)	.93
Total fast walking, min/wk	39.1 (5.1)	44.1 (4.8)	−5.1 (−16.1 to 5.9)	.45	131	39 (5)	136	44.5 (4.7)	−5.5 (−16.2 to 5.2)	.40
Total walking uphill, min/wk	10.3 (2.5)	13.8 (2.5)	−3.6 (−9 to 1.9)	.28	131	11.4 (2.3)	136	14.5 (2.5)	−3.1 (−8.3 to 2.1)	.32
Total errands walking, min/wk	23.7 (6.2)	23.3 (5.2)	0.4 (−12.1 to 12.9)	.96	131	25.1 (6.1)	136	22.4 (5.0)	2.8 (−9.5 to 15.0)	.71
Total leisure walking, min/wk	41.0 (6.8)	32.8 (6.7)	8.3 (−6.2 to 22.7)	.35	131	41.1 (6.7)	136	31.4 (6.5)	9.7 (−4.8 to 24.2)	.27
Change in BMI	−0.2 (0.2)	−0.3 (0.1)	0 (−0.3 to 0.4)	.90	98	−0.2 (0.1)	104	−0.3 (0.1)	0.1 (−0.2 to 0.4)	.57
Change in waist circumference, in	−0.7 (0.4)	−0.4 (0.4)	−0.3 (−0.9 to 0.3)	.43	97	−0.7 (0.5)	100	−0.3 (0.2)	−0.5 (−1.3 to 0.3)	.32
Change in systolic blood pressure, mm Hg	−0.5 (1.0)	−2.3 (1.1)	1.9 (−0.4 to 4.1)	.18	98	−1 (0.9)	104	−3.1 (1)	2.1 (0.1 to 4.1)	.09
Change in diastolic blood pressure, mm Hg	−0.5 (0.7)	−1.4 (0.8)	0.8 (−0.7 to 2.3)	.38	98	−1 (0.6)	104	−1.9 (0.7)	0.9 (−0.6 to 2.3)	.32
Changing in resting heart rate, bpm	−1.8 (1.0)	−1.2 (0.9)	−0.5 (−2.9 to 1.9)	.72	98	−1.5 (0.7)	104	−1.3 (0.8)	−0.3 (−1.8 to 1.3)	.79
Total inactive min/wk	−244.2 (103.9)	−46.0 (141.9)	−198.2 (−436.7 to 40.2)	.17	98	−147.9 (82.4)	104	−29.5 (86.0)	−118.4 (−299.4 to 62.7)	.28
Total inactive TV/screen min/wk	−130.7 (44.3)	−122.6 (53.5)	−8 (−91.3 to 75.2)	.87	98	−108.3 (33.0)	104	−120.4 (26.8)	12.2 (−52.7 to 77.0)	.76
Behavioral variables										
Exercise goal-setting scale^c^	0.89 (0.11)	0.53 (0.08)	0.35 (0.15 to 0.55)	.004	97	0.90 (0.08)	111	0.53 (0.08)	0.37 (0.20 to 0.54)	<.001
Exercise planning and scheduling scale^c^	0.63 (0.09)	0.36 (0.06)	0.27 (0.11 to 0.44)	.01	96	0.63 (0.06)	111	0.36 (0.05)	0.28 (0.15 to 0.41)	<.001
Exercise barrier^d^	0.98 (0.29)	0.37 (0.24)	0.62 (−0.01 to 1.24)	.11	96	0.97 (0.20)	110	0.30 (0.21)	0.67 (0.21 to 1.12)	.02
Physical performance^e^	8.83 (1.59)	6.83 (2.11)	2.01 (−2.44 to 6.45)	.45	94	7.7 (1.22)	104	7.19 (1.24)	0.51 (−2.17 to 3.19)	.75
QoL^f^										
Overall	0.32 (0.11)	0.22 (0.12)	0.10 (−0.15 to 0.35)	.5	97	0.23 (0.08)	113	0.19 (0.07)	0.04 (−0.12 to 0.21)	.68
Rated health overall	0.48 (0.17)	0.54 (0.14)	−0.06 (−0.36 to 0.24)	.73	98	0.13 (0.07)	113	0.05 (0.07)	0.08 (−0.07 to 0.23)	.36
Satisfaction with health	0.16 (0.14)	0.09 (0.12)	0.07 (−0.18 to 0.32)	.63	98	0.47 (0.08)	113	0.50 (0.08)	−0.03 (−0.21 to 0.15)	.76
Subdomains										
Physical health	0.94 (0.20)	0.83 (0.20)	0.12 (−0.30 to 0.53)	.64	98	0.91 (0.17)	113	0.76 (0.17)	0.15 (−0.22 to 0.52)	.5
Psychological	1.29 (0.18)	0.90 (0.20)	0.39 (−0.02 to 0.80)	.12	98	1.25 (0.16)	113	0.86 (0.16)	0.39 (0.04 to 0.73)	.07
Social relationships	0.87 (0.24)	0.17 (0.24)	0.70 (0.12 to 1.27)	.05	98	0.74 (0.22)	113	0.13 (0.21)	0.61 (0.14 to 1.07)	.03
Environment	1.13 (0.17)	0.62 (0.20)	0.51 (0.11 to 0.91)	.04	98	1.07 (0.16)	113	0.64 (0.16)	0.43 (0.09 to 0.77)	.04
Social support for exercise^g^										
Family participation	3.24 (1.08)	−0.16 (0.97)	3.41 (1.20 to 5.62)	.01	97	3.32 (0.89)	110	−0.10 (0.82)	3.41 (1.54 to 5.29)	.003
Family rewards and punishment	0.14 (0.17)	0.10 (0.16)	0.04 (−0.30 to 0.39)	.83	97	0.09 (0.14)	110	0.03 (0.14)	0.06 (−0.25 to 0.37)	.75
Friend participation	1.12 (1.33)	1.74 (1.09)	−0.62 (−2.71 to 1.47)	.63	96	1.06 (0.86)	109	1.21 (0.82)	−0.15 (−2.01 to 1.70)	.89

Abbreviations: BMI, body mass index (calculated as weight in kilograms divided by height in meters squared); MVPA, moderate to vigorous physical activity; PA, physical activity; QoL, quality of life; SMS, short message service.

^a^ Generated from combining the analyses of imputations and mixed-effect models accounting for repeated measures (6 and 12 months) and adjusted for baseline value and the stratifying factor of sex.

^b^ Generated from mixed-effect models with all study collected data accounting for repeated measures (6 and 12 months) and adjusted for baseline value and the stratifying factor of sex.

^c^ Mean of 10-item 5-point Likert scale: 1 to 5; higher score indicates greater agreement.

^d^ Mean of 14-item 10-point Likert scale, range, 0 to 10; higher score indicates more confidence.

^e^ Mean of 16 items; range, 0 to 100; higher score indicates more positive.

^f^ Measured using the World Health Organization quality of life scale and subscales, using a 5-point Likert scale, with 1 indicating not at all; and 5, extremely.

^g^ Measured for family and friends separately; range, 1 to 5; higher score indicates more often.

Both programs significantly increased the proportion of participants reaching or exceeding national MVPA guidelines of at least 150 minutes per week. In the human advisor group, 37 participants (30.8%) achieved the guideline, while 31 participants (24.0%) in the SMS group achieved the guideline (difference, −3.8 [90% CI, −26.7 to 19.1]; 8.3 to −2.8 minutes per week).

Corroborative evidence from the accelerometry data using paired-comparison *t* tests showed that each group achieved significant 12-month increases for median steps per day and median MVPA levels (eResults in Supplement 2). Evaluating between-group differences using linear regression, no significant differences were found for median step per day increases, while the MVPA increase was higher for the human advisor (β = −8.141; SE, 2.609; *P* = .002).

#### Secondary Outcomes

Table 2 summarizes mean changes in reported MVPA, total PA, sedentary time, measured clinical risk factors, well-being, and PA-related behavioral and social strategies. Intention-to-treat analyses indicated similar increases from baseline to 12 months across groups for these PA variables and decreases in inactive minutes involving weekly television or screen time. Within-group descriptive analyses showed significant changes in these variables at 12 months (eTable 1 in Supplement 2). Participants in both groups maintained their weight across 12 months^65,66^ (eTable 1 in Supplement 2).

Both groups reported significant improvements in well-being variables, with similar magnitudes across the physical and psychological health subscales (Table 2). For the social relations and environment subscales, a significantly greater increase was found in the human group (social relationships: difference, 0.7X [90% CI, 0.12 to 1.27]; *P* = .05; environment: difference, 0.51 [90% CI, 0.11 to 0.91]; *P* = .04). Similarly, while both groups reported positive 12-month improvements in relevant behavioral/motivational and social support strategies, the magnitude of the change was significantly greater in the human advisor group for PA goal-setting (difference, 0.35 [90% CI, 0.15 to 0.55]; *P* = .004), PA planning and scheduling (difference, 0.27 [90% CI, 0.11 to 0.44]; *P* = .01), and greater family participation supporting participants’ PA (difference, 3.41 [90% CI, 1.2X to 5.62]; *P* = .01) (Table 2).

### Program Satisfaction and Safety

While at both groups reported general satisfaction and bonding with their advising program at 12 months , the mean rating was significantly higher in the human advisor group (mean [SE] score: human advisor, 6.2 [0.2]; SMS advisor, 5.6 [0.1]; *P* < .001) (eTable 2 in Supplement 2). No serious adverse events or deaths were reported for either group. Milder events included leg, knee, and ankle aches; plantar fasciitis; and shin pain. All participants remained in the study and were able to resume PA following the reported event. SMS technical problems were infrequent and were resolved remotely by CareMessage in partnership with Stanford personnel.

## Discussion

The results of this randomized equivalence trial expand the evidence-based options for PA counseling for midlife and older inactive populations by adding customizable SMS programs as an accessible and effective form of sustained PA advice and support. Based on the evidence-supported Active Choices PA program,^15^ the SMS program was equivalent to the human advisor program in significantly increasing PA outcomes while using substantially less advisor time—a distinct advantage for working adults. The 12-month intervention represents a longer timeframe than previously reported in the mobile health (mHealth) literature, particularly for aging adults and Latino/a populations.^6,13,67^ Both programs also reduced prevalent sedentary behaviors, an independent risk factor for cardiovascular disease and other negative health outcomes.^3^

Study participants’ baseline BMI levels placed them in the class 1 obesity range. While participants did not lose significant weight during the walking program, both study groups maintained stable weight across the 12-month period, unlike the typical yearly weight gain often observed in US adults.^65^

Reported well-being improved in both interventions, with stronger improvements in the human advisor program, consistent with 2 other clinical trials testing human advisors vs other computer-derived PA advice formats.^37,41^ Potential implications of these differences for longer-term PA participation and other behavioral and health outcomes merit continued study.

### Strengths and Limitations

Study strengths include a head-to-head comparison using a rigorous trial design and equivalence hypothesis testing, an aging working population that rarely has been the focus of PA programs and is often underrepresented in clinical trials research,^17^ a longer intervention period than most mHealth PA interventions to date,^13^ solid 12-month retention (>85%) across both groups, and accelerometry measurement that corroborated reported PA.^64^ Significant accelerometer-based increases in steps per day and MVPA occurred in both groups, with additional secondary analysis indicating a larger MVPA increase in the human advisor group vs SMS advisor group. While this difference is intriguing, it is unclear whether the greater increase in the human group was a result of the intervention itself. Given that more than 96% of participants were employed and more than 40% worked in blue-collar jobs which often require increased activity and movement throughout the workday, it is conceivable that the difference could be due to variations in work-related MVPA, which paradoxically has been linked at times, especially in blue-collar workers, to increased cardiovascular health risks.^68,69^ These questions merit further study.

This study has some limitations, including lack of an even longer intervention period (eg, 24 months). While the staff-delivered Active Choices PA intervention has successfully promoted PA maintenance over 18 to 24 months in other studies,^70,71^ no similar studies exist for the SMS advisor that we used. It also is unclear whether results would generalize beyond the midlife and older Latino/a study population. Investigations to further establish the effectiveness of SMS programs customized for other groups are indicated. Such precision intervention approaches can provide more effective solutions than more typical 1-size-fits-all interventions.^14,72^ Given the study’s community-based focus and participants with little experience using wearable PA devices, accelerometers were placed at the hip, which was easier for participants than thigh placement, although the latter can increase sensitivity and specificity in younger adults and athletes.^73,74^ Similar to other clinical trials,^41^ we found hip placement to be sufficient for corroboration of PA increases in both groups.

Additional areas warranting investigation include cost-effectiveness determinations, along with which PA program delivery channels are most attractive and effective for different audience segments, ie, addressing the whiches conundrum.^14^ Prior PA trials using Active Choices have shown success with varied advisor sources, including professional staff,^36,39^ trained community peers and health educators,^38,40,41^ interactive voice response systems,^37,71^ embodied conversational agents,^41,43^ and now, SMS. Study populations also have varied.^36,37,38,39,41,57^

## Conclusions

This randomized equivalence trial found that this behavioral science–informed SMS messaging achieved meaningful 12-month changes in health-enhancing PA in aging adults that was comparable to an established human advisor program. These results are the latest in a programmatic research line aimed at expanding PA program choices and reach in pursuit of advancing population health for all.

## Notes

Supplement 1.
Trial Protocol and Statistical Analysis Plan

Supplement 2. **eResults.** Accelerometry Corroborative Results **eTable 1.** Within-arm Changes, by Arm, at 12 Months for Select Primary and Secondary Outcomes **eTable 2.** Program Advisor Satisfaction Ratings at 12 Months, By arm: Working Alliance Inventory (Bonding Subscale) Results

Supplement 3.
Data Sharing Statement
