# Supplementary material for: Human Telephone vs Text Message Counseling and Physical Activity Among Midlife and Older Adults: A Randomized Clinical Trial
Source: JAMA Netw Open. 2025 Sep 4;8(9):e2528858. doi: 10.1001/jamanetworkopen.2025.28858 (PMC12411977; doi:10.1001/jamanetworkopen.2025.28858)
Supplement: Supplement 1. — Trial Protocol and Statistical Analysis Plan [file jamanetwopen-e2528858-s001.pdf]

ONLINE SUPPLEMENT 1: PROTOCOL[a]

Human Telephone vs Text Message Counseling and Physical Activity among Lower-income Adults: A Randomized Clinical Trial

Abby C. King, PhD et al., Stanford University School of Medicine

[a] Adapted from King AC, Campero MI, Sheats JL, Castro Sweet CM, Rodriguez Espinosa P, Garcia D, Hauser M, Done M, Patel ML, Parikh NM, Corral C, Ahn DK. (2020). Testing the effectiveness of physical activity advice delivered via text messaging vs. human phone advisors in a Latino population: The *On The Move* randomized controlled trial design and methods. *Contemp Clin Trials.* NIHMS ID 1613317; clinicaltrials.gov Identifier # NCT02385591. Available online 11 July 2020.

1. Introduction

Physical inactivity is an important risk factor for a number of chronic diseases, conditions, and outcomes, including premature mortality, cardiovascular disease, eight forms of cancer, and other noncommunicable diseases.[1] Given the decades-long recognition of physical activity (PA) as an independent risk factor for the major noncommunicable diseases and conditions plaguing the U.S.[1] and many other countries worldwide,[2] developing and testing scalable interventions that can significantly increase regular physical activity levels is considered to be a high-priority objective with important clinical implications.

Despite the demonstrated benefits that participation in regular PA can have across the life course, about 50 percent of U.S. adults fall below recommended levels of regular aerobic physical activity (PA).[3] Insufficient PA levels are particularly common among lower-income populations such as Latino adults– a growing segment of the U.S. population– for whom few adapted programs have been developed and tested.[4]

Text messaging (SMS) represents a convenient and accessible communication channel for delivering targeted PA information and support but has not been rigorously evaluated against standard human-delivered telehealth advising programs. Such interactive computer-delivered programs can be significantly more convenient for individuals, given that they do not require ongoing attendance at in-person classes or programs.[5-7] The latest national trends consistently reflect the continuing widespread use and popularity of SMS platforms across virtually all US population segments and sectors, including individuals from different age groups and socioeconomic strata.[8-10] For instance, texting is the top reason why 88% of people use their mobile phones;[10] surveys indicate that texting is the preferred way to communicate (more so than emails, phone calls, and other types of messaging apps), including with businesses;[9,10] text messages have as much as a 98% open rate;[10] and 85% of people check a text within 5 minutes of receiving it.[10] Texting is typically low-cost, easy to use, and convenient, and given this, industry experts predict that AI and similar advanced technology tools will be integrated into text-messaging platforms as opposed to supplanting them.[8]

While the current SMS literature in the PA field suggests that SMS interventions can be useful in increasing physical activity,[11] there are a number of limitations and constraints in this field, including short intervention periods (less than 6 months); small sample sizes (<100 participants total); variability in study quality; frequent inclusion of additional treatment strategies on top of SMS (e.g., educational sessions) which serve to blur the unique impacts of the SMS intervention alone; and general lack of interactivity and customization for the target population, as well as a dearth of individual tailoring in response to individual preferences concerning message frequency, delivery times, etc. Notably, only two of the studies included in a recent review explicitly enrolled Latino/a populations, and both of these studies included a single arm only (i.e., were not RCTs).[11] No study has directly investigated the sustained effectiveness (at the end of a year) of an SMS-delivered PA program in comparison with an established, evidence-supported human advisor-delivered PA program. The latter comparison directly addresses the potential potency of carefully constructed SMS programs when compared to established though more staff-intensive "human touch"-centered alternatives, and in this way directly contribute to expanding the evidence-based choices underlying the goals of "precision behavioral medicine".

The goal of the *On The Move* randomized clinical trial was to test the 12-month equivalence of a novel linguistically and culturally targeted SMS PA intervention (SMS PA advisor) in comparison with an established PA phone advisor intervention delivered by trained staff. The primary hypothesis being tested was the potential equivalence of two PA interventions[12] – an effective evidence-based human phone advisor program demonstrated in multiple clinical trials to be superior to various control arms[13-17] vs. an experimental customized text-messaging advisor program. To date, few SMS interventions have been based on relevant behavioral theory or customized specifically for midlife and older adults, and most such mHealth interventions have been less than 6 months in duration. The *On The Move* Trial represents arguably the first study to systematically compare the effects of a customized physical activity SMS

advising program to a standard, staff-delivered physical activity phone advising program in midlife and older adults of primarily Latin American descent. Additional trial design features aimed at minimizing threats to internal validity included controlling for seasonality impacts on PA through using 12-month change as the primary end point (which occurred during the same season as baseline measurement) and enrolling participants across all four seasons.

2. Methods and Procedures

The Stanford University School of Medicine Institutional Review Board approved the study protocol for this trial. All study materials, including informed consent and recruitment, intervention, and assessment forms, were produced in English and underwent thorough translation into Spanish by certified translators. Participants provided written consent upon reviewing the consent form with a bilingual staff member. The trial was registered at Clinicaltrials.gov (#NCT02385591).

2.1. *Study design*

The major objective of this randomized controlled trial (RCT) was to compare two different communication modalities (standard human telephone advising vs. interactive SMS advising) for delivering a theoretically derived, evidence supported PA telephone advising program called *Active Choices* across 12 months.[15-18] The study design was a randomized parallel group clinical trial testing intervention equivalence using a 1:1 allocation ratio. The study enrolled 280 adults from November 2015–September 2017, with follow-up through October 2018. Data analysis was performed January 2023–December 2024. The primary hypothesis being tested as the main focus of this trial pertained specifically to comparing the potential equivalence of the two PA interventions[12] (an effective evidence-based human phone advisor program demonstrated in multiple clinical trials to be superior to various control arms[13-17] vs. an experimental customized text-messaging advisor program). An additional substantially smaller SMS nutrition arm was included in the trial to explore secondary questions pertaining specifically to the SMS delivery channel alone, and thus is not included in this main trial paper reporting the results relating to the major physical activity equivalence hypothesis being tested.

Eligible participants were recruited from five counties located in the greater San Francisco Bay Area, CA (Santa Clara, San Mateo, Monterey, Alameda, and Santa Cruz Counties). Study eligibility criteria included the following: (a) ages 35 years and older; (b) insufficiently active,[19] i.e., engaged in less than 125 minutes/week of moderate intensity activity such as brisk walking over the past six months, based on initial study physical activity screening items (see below), followed by a final baseline physical activity determination using the Community Healthy Activities Model Program for Seniors (CHAMPS) questionnaire;[20] (c) able to safely engage in moderate forms of physical activities such as walking based on the Physical Activity Readiness Questionnaire (PAR-Q);[21] (d) able to read and understand English or Spanish sufficiently to provide informed consent and participate in all study procedures; e) planning to live in the area for the next twelve months; f) self-reported body mass index (BMI) $\geq$25 and $\leq$46 kg/m^2, which was based on self-reported height and weight; g) not pregnant, planning to become pregnant in the next year or had a newborn in the past year; and h) self-identified as Latino or Hispanic. The study screen to determine physical activity status included the following questions: 1) In the last three months have you regularly participated (at least two times per week) in any physical activity that has increased your breathing, such as aerobics, brisk walking, dancing, swimming, or playing sports? If the participants answered “yes”, they were asked to describe how many days per week and minutes per day they regularly exercised. These numbers were multiplied to calculate the total number of minutes per week. If the total number was 125 minutes or more, the individual was deemed ineligible. If the total number of minutes per week was reported as less than 125, then individuals were asked to report, in a typical or normal week over the past four weeks, the total number of minutes per week they engaged in dance, walking or hiking uphill, walking fast or briskly for exercise, and water exercises other than swimming. Individuals were deemed eligible if participation in the above exercises totals less than 125 minutes per week. Physical

activity patterns were further evaluated at baseline using the full CHAMPS questionnaire.

2.2. *Study participant recruitment and screening methods*

Three complementary recruitment methods were employed to increase external validity, as follows: geographically defined targeted mass mailings, cultural media-based promotion, and community outreach.[22-24] For the geographically defined targeted mass mailings, mailing addresses of residents in geographically defined Census block groups in the five target counties were accessed via a private mail service company and selected based on age and reported Latin American ancestry. Introductory bilingual letters describing the study and business reply cards were sent to households, along with a toll-free number to call and study website link to obtain further study information and undergo initial screening for study eligibility. The media promotional methods included university and other community email lists serving significant numbers of Latino adults and study announcements placed in local Spanish language newspapers and on local Spanish television and radio stations. As part of community outreach activities, study information was placed at local community centers and was made available at local stakeholder events attended by bilingual study staff, such as health and resource fairs, back to school nights, local school council events, as well as local libraries, churches, local health clinics serving significant numbers of Latino adults, and local grocery stores.[25] Study participants and others who expressed interest in the study were encouraged to refer others or share study information with others.

Interested individuals could complete the eligibility screen, which took approximately 15 minutes, via the study website or through a phone call with study staff. If eligible based on the online screen, a staff member contacted the individual to verify eligibility and schedule an orientation session.
Those individuals judged to be initially eligible based on the telephone or web-based screen were invited to attend, depending upon where they lived, a group study orientation session at either Stanford University or a designated community center or similar location in their area. During the study orientation the study objectives and procedures were explained in further detail, all questions were answered, and interested individuals were consented and scheduled for an individual baseline assessment visit held at the same location. Following the baseline assessment, those individuals found to be eligible and willing to enroll in the study were randomized to receive one of the study interventions, followed by an initial introductory session (lasting about 30 minutes) for their designated intervention, typically in person but occasionally conducted by phone with research staff when an in-person meeting was unable to be scheduled. Random sequence allocation was achieved using a computerized version (delivered using the Microsoft Excel application) of the well-established Efron biased coin randomization procedure,[26] which serves to balance the numbers randomly assigned to each arm of an experimental study across the entire study enrollment process. Doing so helps to mitigate any heterogeneity or time trends in subject characteristics that may lead to less comparable treatment arms. A 1:1 allocation ratio by gender was used for the two physical activity arms to help ensure similar numbers of men and women in each of the two arms. The randomization allocation process was performed by a senior staff member not directly involved in study assessments or intervention delivery. Allocation concealment was in place during the randomization process to minimize selection and similar types of allocation bias. Assessment staff members were blinded to randomization assignment and to prior assessment data.

2.3. *Participant retention methods*

To enhance participant understanding of study goals and methods and foster study retention, initial group-based study orientation sessions were employed following the telephone screening process and prior to baseline assessment to ensure that all individuals considering study participation were fully informed about the study objectives and expectations. This type of interactive pre-enrollment educational session has been linked with high levels of study retention in health behavior change trials lasting through 18 months.[27] In addition, modest remuneration for participation in each of the three study assessment points involved a $20/assessment gift card, used as a small “thank you” for assessment completion. There

is no evidence that we are aware of indicating that small remuneration for scientific assessment completion impacts participation levels in the types of behavioral interventions being tested.[28]

2.4. *Study intervention development and delivery: Conceptual overview*

The two physical activity interventions were based on theoretically derived cognitive-behavioral advice and support strategies used in the evidence-based *Active Choices* physical activity counseling program and similar behaviorally based, evidence-supported interventions in the field.[13,16,17,29] The *Active Choices* PA phone intervention has been demonstrated as effective in increasing PA levels in a range of populations across more than three decades of NIH-funded clinical trials.[13-17,30] It is one of the few PA interventions to show effectiveness across periods as long as 18-24 months.[14,31] The repeated success of the *Active Choices* PA phone advising program has led it to be listed on national websites (National Council on Aging, CDC) as an effective evidence-based program for physical activity.

Intervention content for the two arms is summarized in **Table 1** below.

Similar to other evidence-based and supported PA programs, the extensively tested *Active Choices* phone advisor program upon which the two interventions are based, (which has more than 30 years of clinical trial evidence supporting its effectiveness in a range of populations),[13-17,30] is grounded in tested principles of progression with respect to the frequency, intensity, and duration of walking and other PA behaviors of relevance to the target population.[18,32,33] In both interventions, increases in regular physical activity throughout the day was encouraged through progressively increasing PA frequency, intensity and duration, commensurate with current national PA guidelines.[34] While all types of activities were generally encouraged, regular walking was chosen as the primary focus of the interventions for the following evidence- supported reasons: a) Walking remains the most attractive, convenient, accessible, and typically safest form of physical activity for midlife and older populations, especially for more inactive populations such as Latino/a adults.[1,28] b) An expanding amount of evidence supports increasing the overall volume of walking and similar activities in inactive aging adults as a useful means for enhancing overall health and reducing health risks.[1] For example, a 2019 longitudinal study of older US women found that it was the overall volume of steps/walking per day, rather than their intensity, that predicted lower overall mortality rates,[35] with similar results reported in the NIH All Of Us Program analysis showing that overall volume of PA (captured through daily step counts described as walking) remained significantly associated with incident chronic disease outcomes after accounting for the intensity of steps/movement throughout the day.[36] Finally, a recent meta-analysis of prospective cohort studies comparing volume vs. intensity of PA and mortality in midlife and older adults found that total volume of PA was associated to a greater extent with lower mortality risk than MVPA. They concluded that any intensity of PA can lower mortality risk in midlife and older adults.[37]

Thus, the current evidence base among midlife and older adults supports increasing total volume of weekly PA, such as walking, of both moderate intensity and lighter intensity. The walking Items constituting the CHAMPS PA instrument include walking at higher intensity (i.e., at a fast pace; walking uphill) as well as those occurring at a more leisurely pace.

The human phone advisors delivering *Active Choices* in this trial were trained Stanford intervention staff with bachelor or master degrees in health sciences who had relevant experience in delivering the *Active Choices* PA phone intervention. Both intervention arms were provided with a pedometer to help them track their activity amounts, which is considered to be standard practice in current PA behavioral interventions.[28] Providing them to both arms eliminated any differential effects across the two arms.

2.5. *Development of the SMS physical activity intervention*

The PA SMS intervention was developed in collaboration with CareMessage, a not-for-profit organization that builds mobile health solutions for low-income populations.[38-41] Their patented, text

message-based coaching technology enables providers to interact with patients/users through automated messages. Message content was customizable and, for the purposes of this study, could be varied with respect to the frequency and timing of delivery, as follows: following randomization to this arm, participants were asked to choose the frequency with which text messages would be sent to their mobile phones. The frequency categories were as follows:

• Low frequency: 2-3 texts/week.
• Medium frequency: 3-4 texts/week.
• High frequency: 4-5 texts/week.

Among participants randomized to this program (N=141), 20% (N=28) chose low text frequency, 53% (N=75) chose medium text frequency, and 27% (N=38) chose high text frequency.

Following randomization, SMS advisor participants also chose the time of day (morning or afternoon) in which they wanted to receive the text messages. 58% chose mornings and 42% chose afternoons.

Approximately half of the SMS messages (48.6%) sent to each participant across 12 months invited a response, asking the message receiver for answers to specific questions about goals (e.g., how many times per week a participant planned to engage in physical activity).

Prior to initiating the *On The Move* Trial, a pilot study was conducted with CareMessage to test the feasibility and acceptability of SMS message content with 30 inactive Latino adults who met *On the Move* study eligibility. Flesch-Kincaid grade level for the messages[42,43] averaged 3.8 (range 1.2-5.8), which indicated that average readability was between third and fourth grade education levels. All of the messages the system sent were delivered successfully, and 88% reported that they would recommend the SMS advisor to friends and family.

**Table 1.** Intervention Content	**Human PA Telephone Advisor**	**Interactive SMS PA Advisor**
Program Contact	• Initial face-to-face session (30mins)<br>○ PA Recommendations given<br>○ Initial Goal set by Participant<br>○ Pt given pedometer, logs, resource packet, program calendar<br>• 10-24 calls scheduled (based on participant preferences, with flexibility to increase/decrease as needed)<br>• Approx. 15 minutes per call<br>• Periodic tip sheets mailed as requested	• Initial SMS Advisor intro session (30 mins)<br>○ PA Recommendations given<br>○ Customizing info collected from participant (SMS frequency, time of day)<br>○ Initial goal set by Participant<br>○ Pt given pedometer, logs, resource packet, program calendar<br>• 2-5 texts sent per week (with flexibility to increase/decrease)<br>• Texting frequency maintained throughout trial<br>• Approx. 1 minute or less read/response time per text (based on pilot-testing of the SMS system with staff and community members that were part of the study target population)
Self-Regulation:<br>• Self-monitoring<br>• Goal setting<br>• Personalized feedback<br>• Knowledge enhancement	• Participant provided tools to self-monitor PA<br>• Participant self-reports goals & activity achieved (e.g., minutes)<br>• Advisor verbalizes personalized feedback (achieved, came close, exceeded goal)	• Participant provided tools to self-monitor PA<br>• Participant text-enters goals (active days & minutes) and activity achieved (e.g., minutes)<br>• Personalized feedback texted on performance (achieved, came close, exceeded goal)
Social Support:<br>• Enlist friends/ family<br>• Support from advisor<br>• Support-seeking ability	• Participant asked to identify role models & support persons<br>• Norms for physical activity discussed<br>• Participant advised to cultivate supportive relationships with important others<br>• Advisor cultivates supportive/nurturing relationship to participant	• Participant asked to think of role models & support people<br>• Texts periodically encourage user to check in with role models & support people<br>• Prompts on family values around PA texted<br>• Texts relaying supportive/nurturing encouragement
Autonomous Motivation:<br>• User drives choices<br>• User determines goals	• Participant determines initial goals<br>• Participant verbally negotiates to increase/decrease or maintain goals<br>• Participant can request more/less contact<br>• Participant difficulties verbally acknowledged	• Participant determines initial goals<br>• Participant texts to increase, decrease or maintain goals<br>• Participant texts to receive more/less contact<br>• Participant difficulties acknowledged by text

2.6. *Intervention fidelity and quality assurance*

For the human PA telephone advisor arm, trained staff members conducted regular quality control checks and activities as has been standard with the *Active Choices* telephone advising program.[30,44] These activities included completion of a structured call checklist/log which consisted of number and dates of telephone call attempts and completions, and number of minutes per contact for each completed phone contact. A supervisor-led intervention team meeting occurred on a weekly basis throughout the project intervention period to discuss any intervention-related challenges that occurred during the week, and intervention-related questions and issues were brought to the project principal investigator – a trained clinical psychologist – who provided advice and support in ensuring the effective handling of the intervention contacts. These activities were accompanied by regular review by senior intervention staff of the telephone advisor checklist/logs and notes completed after each advisor-participant session, along with timely review of the audiotapes that were collected (with participant consent) for all advising sessions. Finally, random check-ins by senior intervention staff with study participants occurred as an additional method of ensuring intervention fidelity and quality.

Quality assurance for the SMS advisor arm included regular monitoring of system performance and backup, participant response rates, and ongoing availability of a study helpline for participants to call for assistance in correcting any problems. A designated Stanford staff member was in regular contact (twice a month or more frequently as needed) with the CareMessage Product team to ensure that any problems that occurred could be resolved in a timely manner.

The weekly intervention staff meetings throughout the study period allowed intervention staff and supervisors to engage in mutual sharing and problem-solving of their experiences and challenges with their participants of relevance to ongoing intervention engagement and study retention.

2.7. *Assessment: Primary outcome*

The primary outcome was change in total weekly walking minutes across the 12-month intervention period—which represents the most appealing and easy to do physical activity in this age group.[1] For all participants, physical activity was assessed at three time points: baseline, an interim assessment at six months, and a full assessment at 12 months, with the 12-month assessment considered the primary endpoint. To provide specific information on physical activity types, the validated Community Healthy Activities Model Program for Seniors (CHAMPS) questionnaire for midlife and older adults (interview format),[20,45] available in English and Spanish, was used to assess usual weekly minutes of different types and intensities of PA over the previous 4 weeks. Such validated self-report instruments represent the most direct and reliable means for assessing the specific types of PA typically being targeted in interventions, given that device-based assessment tools (pedometers, accelerometers) capture more general movement levels beyond such purposeful PA behavior.[46,47] CHAMPS variables have been consistently associated with device-based physical activity measures in prior studies,[48,49] and the CHAMPS has been reported to be the only PA instrument for older adults that correlates with both doubly-labeled water-measured PA energy expenditure and accelerometers.[50] It also has been shown to be sensitive to change with moderate-intensity PA programs such as *Active Choices* in a variety of community samples, including Latino adults.[7,16,17,30]

Use of the CHAMPS instrument to capture total volume of walking behavior was chosen for the following additional reasons:

a) this psychometrically valid and reliable instrument was explicitly developed to capture data on the types of physical activity behaviors being specifically targeted in behavioral intervention studies of community-based aging adults, in contrast to device-based tools which measure general movement but do not provide information on the types of activities being undertaken and how they may be differentially affected by the interventions being tested. It also has been shown to be particularly effective in reliably assessing PA changes in individuals with lower income and educational attainment.[51] Because of these strengths, a number of major PA trials with aging adults have used the CHAMPS instrument as the principal PA measuring tool to capture walking and other types of PA (e.g., the LIFE multi-site Trial).[45] The reliability, validity, and sensitivity of the CHAMPS instrument were optimized in this study through

employing it in its standardized interview format, which was used successfully in the large multi-site LIFE PA Trial with aging adults.[45]

b) CHAMPS is among the limited number of PA instruments for aging adults demonstrated across diverse populations to be sensitive to change, including midlife and older Latino/a adults, for whom there are few PA intervention studies, and even fewer using device-based measurement as the major outcome. There therefore are an insufficient amount of normative data based on devices to aid data interpretation in this population.[7,16,17,30,33] In contrast, the 70-year evidence base linking physical activity to a range of important health outcomes has been driven primarily by systematically collected self-report data.

c) Researchers have found at times that accelerometry can underestimate movement and may be less sensitive to change in older and relatively inactive populations,[50] which can be due to slow walking pace, gait issues, participant device placement problems in this type of community-based study, and similar issues. This has led an increasing number of researchers to recommend combining self-report and device-measured PA.[47,52]

d) In prior community-based PA intervention studies with midlife and older inactive Latino/a adults, participants often have noted the devices to be burdensome to wear,[7] especially in light of the conditions of their daily activities, including work, caregiving, and related activities. In the current study, >96% of participants were employed outside the home, many having more than one job throughout the week, with >40% employed as blue-collar workers with unpredictable work schedules and locations. Therefore, similar to prior studies with this population, wearing such devices across a week has proven onerous in this lower-income, working, and aging population.[7]

The proportion of each study arm meeting the national physical activity recommendations of at least 150 minutes/week of moderate-to-vigorous physical activity also was evaluated.[53]

2.8. *Secondary outcome measures*

Secondary physical activity outcomes of interest from the CHAMPS included moderate-to-vigorous physical activity (MVPA) and total physical activity levels. Physical activity measurement using the CHAMPS was accompanied by the validated Actigraph® accelerometer (model wGT3X) as a secondary, corroborative activity measure.[54] The accelerometry protocol from a large study of 860 older adults was applied.[46] The activity monitor was worn on the hip during waking hours for seven consecutive days at each time point, ensuring a sufficient number of days of physical activity data (at least four days is considered as complete data) commensurate with current physical activity studies in older adults.[55] Participants were instructed to wear the accelerometer for at least eight hours per day during their waking hours. Wear-time validity was determined through applying the wear and non-wear time analysis and classification algorithms reported by Choi et al.,[56] and analysis and interpretation of the accelerometry data were based on our prior investigations and those of other aging adult populations,[57] including Copeland et al.[58]

Other secondary outcome variables of relevance to aging Latino populations included the following: sedentary/inactive recreation behaviors, measured using a validated one-week recall survey responsive to change in older adults;[59] body mass index (BMI), derived using standard clinical assessment protocols for height and weight;[45] abdominal adiposity through waist circumference and resting blood pressure and heart rate, using standard protocols;[45] behavioral and social support strategies shown to promote positive PA change;[28] and quality of life and well-being, measured with the 26-item World Health Organization (WHO) Quality of Life and self-rated health questionnaire (brief version),[60,61] which assesses perceived health and well-being related to four domains--physical health, psychological health, social relationships, and the environment. Participant program acceptability ratings for the SMS and human advisor phone interventions were assessed at 12 months using the Working Alliance Inventory’s 12-item bonding subscale.[7,62] Sociodemographic information was collected using standard questionnaires.[13] Program safety and adverse events were tracked at each assessment time point with standardized forms and protocols used in prior PA and other health promotion intervention trials.[17,30]

2.9. *Sample size calculation and data analysis plan*

Sample size estimates were developed to test the study's primary question related to whether the automated SMS physical activity advisor intervention resulted in 12-month weekly walking increases that were similar to those achieved in the human physical activity advisor intervention. A final conservative sample size of 280 (~140 participants per PA arm) was established to provide greater than 80% power to determine 12-month equivalence of the two PA arms assuming 15% study attrition rates.[63] Based on the literature,[1] a clinically meaningful difference between arms of 30 min of total walking minutes per week with an estimated within-arm standard deviation of 100 (Cohen's *d* effect size of 0.30) was used to determine study sample size. The software used for sample size calculations was Sealed Envelope Ltd, 2012. The above equivalence margin for the two interventions (a difference in 12-month PA change between arms, in either direction, within 30 mins/week) is based on previous studies performed with *Active Choices* showing significant differences of more than this between the *Active Choices* intervention and non-PA/attention control arms,[12,16,17,30] in addition to literature indicating that with increasing episodes of physical activity/week – with such episodes typically lasting about 30 minutes[16,64] – risk of all-cause mortality, CVD, and other chronic disease outcomes decrease.[1,65] The use of two-sided 90% confidence intervals (CI) are the standard method for determining equivalence in intervention research.[66] In such equivalence procedures, the TOST procedure (two one-sided tests) is used to determine whether the difference in intervention means falls within the stated boundaries that are set up front. In any one-sided test, for an alpha level of .05, one can reject Ho when the 90% CI around the observed estimate is in the predicted direction and does not contain the value the estimate is being tested against. In the TOST procedure, the first one-sided test is used to test the estimate against values at least as extreme as the lower equivalence bound, and the second one-sided test is used to test the estimate against values at least as extreme as the upper equivalence bound. Even though the TOST procedure consists of two one-sided tests, it is not necessary to control for multiple comparisons because both tests need to be statistically significant for the researcher to draw a conclusion of statistical equivalence. Consequently, when reporting an equivalence test, it suffices to report the one-sided test with the smaller test statistic and thus the larger p value.

To account for missing data, multiple imputations were performed by replacing missing 12-month values with a collection of credible values using the option of imputation by fully conditionable specification methods.[67,68] Specifically, the missing outcomes were imputed using fully conditional specification (FCS) methods. FCS methods use appropriate regression models (e.g. linear regression for continuous variables, logistic regression for categorical variables) to sequentially impute missing values based on all other observed/imputed variables as predictors."[69] The algorithm for FCS was implemented in SAS procedure PROC MI,[70] as follows: 10 imputed data sets were created, with each imputation analyzed using PROC MIXED and then PROC MIANALYZE, which combined 10 sets into final estimates. The variables for the imputations included treatment assignment, socio-demographics (e.g., gender, age, job type, marital status, education, employment, household size, and race/ethnicity), baseline values of the outcome, and 6 and 12 month data for each outcome. The fixed effects of each model included baseline value of the outcome, arm (Human advisor or SMS advisor), time point (6 or 12 months), and group × time interactions. The random effects accounted for repeated measures on each participant.

All reported outcomes were based on intention-to-treat principles. Change in 12-month walking minutes per week was the primary outcome, with adjusted coefficients and two-sided 90% confidence intervals generated which accounted for baseline PA value and the stratifying variable of sex and included 6- and 12-month data. Using a two-sided 90% CI constructed for the between-arm difference in the primary outcome, equivalence was deemed to be established if the adjusted estimate lay within the equivalence margins of ±0.30,[71,72] which is considered, based on the evidence described above, to be a meaningful increase in PA to enhance health.[1,65,73] When evaluating equivalence testing, if the entire 90% confidence interval falls within the equivalence margin, the conclusion can be drawn that the two interventions are equivalent. The primary equivalence hypothesis was tested using a mixed-effects linear

regression model that incorporated 6- and 12-month data (SAS v9.4, 2022).[74] Change in total walking minutes/week over 12 months was the primary outcome, with adjusted coefficients accounting for baseline PA value and the sex stratification variable. (Age, evaluated in an initial model, was nonsignificant.) For completeness, both intention-to-treat and completer analyses were reported. Similar analyses described above were conducted for secondary variables of interest. Within-arm pre- and post *t* tests and between arm 12-month testing of specific secondary outcomes (e.g., program satisfaction) were conducted for descriptive purposes. The significance threshold was alpha= .05 using 2-tailed tests. Baseline characteristics were summarized using descriptive statistics including independent-sample *t* tests for continuous variables and Chi-square tests for categorical variables.

In addition, accelerometry-based corroboratory analyses were conducted[49] based on intention-to-treat principles and taking account of data skewness in this sedentary sample. Between-arm baseline to 12-month changes in the primary accelerometry variables (i.e., median steps/day; moderate-to-vigorous physical activity [MVPA]) were evaluated using linear regressions, and paired comparison t-tests were used to evaluate within-arm changes in baseline to12-month accelerometry-measured steps/day and MVPA. (See eSupplement2).

REFERENCES

1. Physical Activity Guidelines Advisory Committee. *2018 Physical Activity Guidelines Advisory Committee Scientific Report*. 2018.
2. World Health Organization. *World Health Organization 2020 guidelines on physical activity and sedentary behavior*. 2020. https:// bjsm. bmj. com/ content/ 54/ 24/1451.
3. U.S. Department of Health and Human Services. *Physical Activity Guidelines for Americans, 2nd edition*. 2018.
4. Pleis JR, Ward BW, Lucas JW. *Summary health statistics for U.S. adults: National Health Interview Survey, 2009*. Vol. 10(249). 2010. *Vital Health Stat*.
5. Silvia CA. Barriers to physical activity in the Hispanic community. *J Public Health Policy*. 2003;24(1):41-58.
6. Marquez DX, Hoyem R, Fogg L, Bustamante EE, Staffileno B, Wilbur J. Physical activity of urban community-dwelling older Latino adults. *J Phys Act Health*. Sep 2011;8 Suppl 2:S161-70.
7. King AC, Bickmore TW, Campero MI, Pruitt LA, Yin JL. Employing "virtual advisors" in preventive care for underserved communities: Results from the COMPASS study. *J Health Commun Int Perspect*. 2013;18(12):1449-64. doi:10.1080/10810730.2013.798374
8. Olia A. Text Messaging Statistics & Trends for 2024 and Beyond. Intradyn.com. Accessed April 16 2025,
9. Weiche A. Survey: Texting is the Preferred Way to Communicate. Leadferno. April 16 2025, http://leadferno.com/blog/survey-texting
10. SlickText. 44 mind-blowing SMS marketing and texting statistics. SlickText. Updated March 12, 2025. Accessed April 16, 2025,
11. Smith DM, Duque L, Huffman JC, Healy BC, Celano CM. Text message interventions for physical activity: a systematic review and meta-analysis. *Am J Prev Med*. January 2020 2020;58(1):142-151. doi:10.1016/j.amepre.2019.08.014
12. Gomberg-Maitland M, Frison L, Halperin J. Active-control clinical trials to establish equivalence or noninferiority: Methodological and statistical concepts linked to quality. *Am Heart J*. 2003;146(3):398-403. doi:10.1016/S0002-8703(03)00324-7
13. King AC, Haskell WL, Taylor CB, Kraemer HC, DeBusk RF. Group- vs home-based exercise training in healthy older men and women. A community-based clinical trial. *JAMA*. Sep 18 1991;266(11):1535-42.
14. King AC, Haskell WL, Young DR, Oka RK, Stefanick ML. Long-term effects of varying intensities and formats of physical activity on participation rates, fitness, and lipoproteins in men and women aged 50 to 65 years. *Circulation*. May 15 1995;91(10):2596-604.
15. King AC, Baumann K, O'Sullivan P, Wilcox S, Castro C. Effects of moderate-intensity exercise on physiological, behavioral, and emotional responses to family caregiving: a randomized controlled trial. *Journal of Gerontology: Medical Sciences*. 2002;57A:M26-M36.
16. King AC, Friedman RM, Marcus BH, et al. Ongoing physical activity advice by humans versus computers: The Community Health Advice by Telephone (CHAT) Trial. *Health Psychol*. 2007;26:718-727.
17. King AC, Castro CM, Buman MP, Hekler EB, Urizar G, Ahn DG. Behavioral impacts of sequentially versus simultaneously delivered dietary plus physical activity interventions: The CALM Trial. *Ann Behav Med*. 2013;46(2):157-68. doi:10.1007/s12160-013-9501y
18. Castro CM, King AC. Telephone-assisted counseling for physical activity. *Exerc Sport Sci Rev*. Apr 2002;30(2):64-8.
19. Physical Activity Guidelines Advisory Committee. *Report of the Physical Activity Guidelines Advisory Committee, 2008*. 2008. DHHS website <http://health.gov/paguidelines>
20. Stewart AL, Mills KM, King AC, Haskell WL, Gillis D, Ritter PL. CHAMPS physical activity questionnaire for older adults: outcomes for interventions. *Med Sci Sports Exerc*. Jul 2001;33(7):1126-41.

21. Thomas S, Reading J, Shephard RJ. Revision of the Physical Activity Readiness Questionnaire (PAR-Q). *Canadian J Sports Sci*. 1992;17:338-345.
22. King AC, Harris RB, Haskell WL. Effect of recruitment strategy on types of subjects entered into a primary prevention clinical trial. *Annals of Epidemiology*. 1994;4:312-320.
23. Kiernan M, Phillips K, Fair JM, King AC. Using direct mail to recruit Hispanic adults into a dietary intervention: an experimental study. *Ann Behav Med*. Winter 2000;22(1):89-93.
24. Brown SD, Lee K, Schoffman DE, King AC, Crawley LM, Kiernan M. Minority recruitment into clinical trials: Experimental findings and practical implications. *Contemp Clin Trials*. Jul 2012;33(4):620-3. doi:S1551-7144(12)00079-1 [pii] 10.1016/j.cct.2012.03.003
25. Soto J, Campero MI, Castro CM, King AC. Lessons from recruiting Latino older adults into the LIFE trial. presented at: Society of Behavioral Medicine 33rd Annual Meeting and Scientific Sessions: Rapid Communications; April 13, 2012 2012; New Orleans, LA. http://www.sbm.org//UserFiles/file/am12_rapid-com_handout_LO.pdf
26. Efron B. Forcing a sequential experiment to be balanced. *Biometrika*. 1971;58:403-417.
27. Goldberg JH, Kiernan M. Innovative techniques to address retention in a behavioral weight-loss trial. *Health Educ Res*. Aug 2005;20(4):439-47. doi:cyg139 [pii] 10.1093/her/cyg139
28. King AC, Whitt-Glover MC, Marquez DX, et al. Physical Activity Promotion: Highlights from the 2018 Physical Activity Guidelines Advisory Committee Systematic Review. *Med Sci Sports Exerc*. Jun 2019;51(6):1340-1353. doi:10.1249/MSS.0000000000001945
29. Wilcox S, Dowda M, Leviton LC, et al. Active for Life: final results from the translation of two physical activity programs. *Am J Prev Med*. Oct 2008;35(4):340-51. doi:S0749-3797(08)00605-3 [pii] 10.1016/j.amepre.2008.07.001
30. Castro CM, Pruitt LA, Buman MP, King AC. Physical activity program delivery by professionals versus volunteers: The TEAM randomized trial. *Health Psychol*. May 2011;30(3):285-94. doi:201109497-006 [pii] 10.1037/a0021980
31. King AC, Hekler EB, Castro CM, et al. Exercise advice by humans versus computers: maintenance effects at 18 months. *Health Psychol*. Feb 2014;33(2):192-6. doi:10.1037/a0030646
32. King AC, Castro C. *Active Choices: Telephone-Assisted Counseling for Physical Activity Manual*. Stanford Prevention Research Center; 2005:1-83.
33. King AC, Campero MI, Sheats JL, et al. Effects of Counseling by Peer Human Advisors vs Computers to Increase Walking in Underserved Populations: The COMPASS Randomized Clinical Trial. *JAMA internal medicine*. Sep 28 2020;180(11):1481-1490. doi:10.1001/jamainternmed.2020.4143
34. Piercy KL, Troiano RP, Ballard RM, et al. The Physical Activity Guidelines for Americans. *JAMA*. Nov 20 2018;320(19):2020-2028. doi:10.1001/jama.2018.14854
35. Lee IM, Shiroma EJ, Kamada M, Bassett DR, Matthews CE, Buring JE. Association of Step Volume and Intensity With All-Cause Mortality in Older Women. *JAMA internal medicine*. May 29 2019;179(8):1105-1112. doi:10.1001/jamainternmed.2019.0899
36. Master H, Annis J, Huang S, et al. Association of step counts over time with the risk of chronic disease in the AllofUs Research Program. *Nature Medicine*. 2022;28(November):2301-2308. doi:10.1038/s41591-022-02012-w
37. Tarp J, Dalene KE, Faagerland MW, et al. Physical activity volume, intensity, and mortality: Harmonized meta-analysis of prospective cohort studies. *Am J Prev Med*. 2024;doi:10.1016/j.amepre.2024.07.022
38. Watterson JL, Rodriguez HP, Shortell SM, Aguilera A. Improved Diabetes Care Management Through a Text-Message Intervention for Low-Income Patients: Mixed-Methods Pilot Study. *JMIR Diabetes*. Oct 30 2018;3(4):e15. doi:10.2196/diabetes.8645
39. Axley P, Kodali S, Kuo YF, et al. Text messaging approach improves weight loss in patients with nonalcoholic fatty liver disease: A randomized study. *Liver Int*. May 2018;38(5):924-931. doi:10.1111/liv.13622

40. Weiss C, Ammerman SD. Designing, implementing and assessing a novel text-messaging intervention for an adolescent mobile health clinic: A collaborative approach. *J Child Adolesc Behav*. 2016;4:299. doi:10.4172/2375-4494.1000299
41. Cheyne K, Smith M, Felter EM, et al. Food Bank-Based Diabetes Prevention Intervention to Address Food Security, Dietary Intake, and Physical Activity in a Food-Insecure Cohort at High Risk for Diabetes. *Prev Chronic Dis*. Jan 9 2020;17:E04. doi:10.5888/pcd17.190210
42. Cotugna N, Vickery CE, Carpenter-Haefele KM. Evaluation of literacy level of patient education pages in health-related journals. *J Community Health*. Jun 2005;30(3):213-9. doi:10.1007/s10900-004-1959-x
43. Williamson JM, Martin AG. Analysis of patient information leaflets provided by a district general hospital by the Flesch and Flesch-Kincaid method. *Int J Clin Pract*. Dec 2010;64(13):1824-31. doi:10.1111/j.1742-1241.2010.02408.x
44. King AC, Sallis JF, Dunn AL, et al. Overview of the Activity Counseling Trial (ACT) intervention for promoting physical activity in primary care settings. *Medicine and Science in Sports and Exercise*. 1998;30:1086-1096.
45. Pahor M, Guralnik JM, Ambrosius WT, et al. Effect of structured physical activity on prevention of major mobility disability in older adults: The LIFE Study Randomized Clinical Trial. *JAMA*. May 27 2014;311(23):2387-96. doi:10.1001/jama.2014.5616
46. King AC, Sallis JF, Frank LD, et al. Aging in neighborhoods differing in walkability and income: Associations with physical activity and obesity in older adults. *Soc Sci Med*. 2011;73:1525-1533. doi:10.1016/j.scoscimed.2011.08.032, http://dx.doi.org/10.1016/j.socscimed.2011.08.032
47. Troiano RP, McClain JJ, Brychta RJ, Chen KY. Evolution of accelerometer methods for physical activity research. *Br J Sports Med*. Jul 2014;48(13):1019-23. doi:10.1136/bjsports-2014-093546
48. Harada ND, Chiu V, King AC, Stewart AL. An evaluation of three self-report physical activity instruments for older adults. *Medicine and Science in Sports and Exercise*. 2001;33:962-970.
49. Hekler EB, Buman MP, Haskell WL, et al. Reliability and validity of CHAMPS self-reported sedentary to vigorous intensity physical activity in older adults. *J Phys Act Health*. 2012;9:225-236.
50. Colbert LH, Matthews CE, Havighurst TC, Kim K, Schoeller DA. Comparative validity of physical activity measures in older adults. *Med Sci Sports Exerc*. May 2011;43(5):867-76. doi:10.1249/MSS.0b013e3181fc7162
51. Resnicow K, McCarty F, Blissett D, Wang T, Heitzler C, Lee RE. Validity of a modified CHAMPS physical activity questionnaire among African-Americans. *Med Sci Sports Exerc*. Sep 2003;35(9):1537-45.
52. Shimoda T, Tomida K, Nakajima C, Kawakami A, Shimada H. Combined self-reported and device-measured physical activity assessment and disability incidence in older adults. *J Am Med Directors Assoc*. June 2025 2024;26(6)doi:10.1016/j.jamda.2024.105375
53. Piercy KL, Troiano RP, Ballard RM, et al. The Physical Activity Guidelines for Americans. *JAMA*. 2018;doi:10.1001/jama.2018.14854
54. Feito Y, Bassett DR, Thompson DL. Evaluation of activity monitors in controlled and free-living environments. *Med Sci Sports Exerc*. 2011;44(4):733-41. doi:10.1249/MSS.0b013e3182351913
55. Hart TL, Swartz AM, Cashin SE, Strath SJ. How many days of monitoring predict physical activity and sedentary behaviour in older adults? *Int J Behav Nutr Phys Act*. 2011;8:62-68.
56. Choi L, Liu Z, Matthews CE, Buchowski MS. Validation of accelerometer wear and nonwear time classification algorithm. *Med Sci Sports Exerc*. Feb 2011;43(2):357-64. doi:10.1249/MSS.0b013e3181ed61a3
57. Rejeski WJ, Marsh AP, Brubaker PH, et al. Analysis and interpretation of accelerometry data in older adults: The LIFE Study. *J Gerontol A Biol Sci Med Sci*. Apr 2016;71(4):521-8. doi:10.1093/gerona/glv204
58. Copeland JL, Esliger DW. Accelerometer assessment of physical activity in active, healthy older adults. *J Aging Phys Act*. Jan 2009;17(1):17-30.

59. Gardiner PA, Clark BK, Healy GN, Eakin EG, Winkler EAH, Owen N. Measuring older adults' sedentary time: reliability, validity, and responsiveness. *Med Sci Sports Exerc*. 2011;43(11):2127-2133.
60. World Health Organization. Development of the World Health Organization WHOQOL-BREF quality of life assessment. The WHOQOL Group. *Psychol Med*. May 1998;28(3):551-8. doi:10.1017/s0033291798006667
61. Skevington SM, Lotfy M, O'Connell KA, Group. W. The World Health Organization's WHOQOL-BREF quality of life assessment: psychometric properties and results of the international field trial. A report from the WHOQOL group. *Qual Life Res*. Mar 2004;13(2):299-310. doi:10.1023/B:QURE.0000018486.91360.00
62. Horvath A, Greenberg L. Development and validation of the Working Alliance Inventory. *J Counseling Psychol*. 1989;36(2):223-233.
63. King AC, Campero I, Sheats JL, et al. Testing the effectiveness of physical activity advice delivered via text messaging vs. human phone advisors in a Latino population: The On The Move randomized controlled trial design and methods. *Contemp Clin Trials*. Aug 2020;95:106084. doi:10.1016/j.cct.2020.106084
64. Physical Activity Guidelines Advisory Committee. *Physical Activity Guidelines Advisory Committee Report, 2008*. 2008. http://www.health. gov/paguidelines/committeereport.aspx.
65. Kushi LH, Fee RM, Folsom AR, Mink PJ, Anderson KE, Sellers TA. Physical activity and mortality in postmenopausal women. *JAMA*. 1997;277:1287-1292.
66. Lakens D, Scheel AM, Isager PM. Equivalence testing for psychological research: A tutorial. *Advances in Methods and Practices in Psychological Science*. 2018;1(2):259-269. doi:10.1177/2515245918770963
67. Inc. SI. *SAS/STAT User's Guide: Version 15.1*. SAS Institute, Inc.; 2018.
68. Little RJA, Rubin DB. *Statistical Analysis with Missing Data, Second Edition*. John Wiley & Sons; 2002.
69. Van Buuren S, Brand JPL, Groothuis-Oudshoorn CGM, Rubin DB. Fully conditional specification in multivariate imputation. *J Statistical Computation and Simulation*. 2006;76(12):1049-1064. doi:10.1080/10629360600810434
70. SAS Institute I. *SAS software, Version 9.4 of the SAS System for Windows*. SAS Institute Inc. ; 2022.
71. Shtaynberger J, Bar H. *Equivalence Testing*. 2023. January 2023. https://cscu.cornell.edu/wp-content/uploads/equiv.pdf
72. Steele RM, Mummery WK, Dwyer T. A comparison of face-to-face or Internet-delivered physical activity intervention on targeted determinants. *Health Educ Behav*. December 2009 2009;36(6):1051-1064. doi:10.1177/1090198109335802
73. Sundquist K, Qvist J, Sundquist J, Johansson SE. Frequent and occasional physical activity in the elderly: a 12-year follow-up study of mortality. *Am J Prev Med*. Jul 2004;27(1):22-7.
74. SAS Institute Inc. *SAS/STAT 9.2 User's Guide, second edition*. SAS Institute Inc.; 2009.
