# Supplementary material for: Human Telephone vs Text Message Counseling and Physical Activity Among Midlife and Older Adults: A Randomized Clinical Trial
Source: JAMA Netw Open. 2025 Sep 4;8(9):e2528858. doi: 10.1001/jamanetworkopen.2025.28858 (PMC12411977; doi:10.1001/jamanetworkopen.2025.28858)
Supplement: Supplement 2. — eResults. Accelerometry Corroborative Results eTable 1. Within-arm Changes, by Arm, at 12 Months for Select Primary and Secondary Outcomes eTable 2. Program Advisor Satisfaction Ratings at 12 Months, By arm: Working Alliance Inventory (Bonding Subscale) Results [file jamanetwopen-e2528858-s002.pdf]

Supplemental Online Content

King AC, Campero MI, Rodriguez Espinosa P, et al. Human telephone vs text message counseling and physical activity among midlife and older adults: a randomized clinical trial. *JAMA Netw Open*. 2025;8(8):e2528858.
doi:10.1001/jamanetworkopen.2025.28858

eResults. Accelerometry Corroborative Results
eTable 1. Within-arm Changes, by Arm, at 12 Months for Select Primary and Secondary Outcomes
eTable 2. Program Advisor Satisfaction Ratings at 12 Months, By arm: Working Alliance Inventory (Bonding Subscale) Results

This supplemental material has been provided by the authors to give readers additional information about their work.

© 2025 King AC et al. *JAMA Network Open*.

eResults: Accelerometry Corroborative Results

- Within-arm differences in accelerometry variable changes from baseline to 12 months (using paired-comparison t-tests):

  Median steps/day change, human advisor arm: Mean Difference = 1,117.51, $t = 9.77$, $p < 0.0001$.

  Median steps/day change, SMS advisor arm: Mean Difference = 879.65, $t = 8.23$, $p < 0.0001$.

  Median MVPA[a] change, human advisor arm: Mean Difference = 5.69, $t = 4.99$, $p < 0.0001$.

  Median MVPA change, SMS advisor arm: Mean Diff = 3.65, $t = 3.33$, $p = 0.0009$.

- Between-arm differences in accelerometry variable changes from baseline to 12 months (using linear regression):

  Median steps/day change comparison: B[b] = -130.112, SE = 417.135, $p = 0.76$.

  Median MVPA change comparison: B = -8.141, SE = 2.609, $p = 0.002$ (increase greater in the human advisor arm).

---

[a] MVPA = Moderate-to-vigorous physical activity

[b] B = Beta estimate

Methods Summary:

The accelerometry protocol from a large study of 860 older adults was applied.[1] The activity monitor was worn on the hip during waking hours for seven consecutive days at each time point, ensuring a sufficient number of days of physical activity data commensurate with current physical activity studies in older adults.[2] Participants were instructed to wear the accelerometer for at least eight hours per day during their waking hours, commensurate with other studies aimed at expanding data inclusivity, including in underserved populations.[3,4]

© 2025 King AC et al. *JAMA Network Open*.

eTable 1: Within-arm Changes, by Arm, at 12 Months for Selected Primary and Secondary Outcomes

	**Human Advisor (n=139)**			**SMS Advisor (n=141)**		
	**Mean Change**	***t* Score**	***P* Value**	**Mean Change**	***t* Score**	***P* Value**
[a] **BP, HR, BMI**						
Systolic BP	-1.00	-0.88	0.38	-3.29	-2.29	**0.03**
Diastolic BP	-0.17	-0.16	0.87	-2.2	-1.94	0.06
Resting HR	-2.33	-1.97	**0.05**	-1.58	-1.24	0.22
BMI	-0.22	-1	0.32	-0.38	-2.12	**0.04**
**CHAMPS PA**[b]						
Walking Min[c]	111.03	23.58	**<0.0001**	102.44	26.09	**<0.0001**
MVPA[d] Min	104.39	28.52	**<0.0001**	87.39	25.64	**<0.0001**
Total PA Min	120.47	12.58	**<0.0001**	136.46	16.50	**<0.0001**
**Exercise Behavior**						
Goal Setting	0.88	108.45	**<0.0001**	0.57	75.50	**<0.0001**
Planning	0.77	113.40	**<0.0001**	0.38	63.53	**<0.0001**
Barriers	1.25	57.98	**<0.0001**	0.01	0.54	0.59
Performance	7.62	54.87	**<0.0001**	4.79	32.83	**<0.0001**
**Physical Inactivity**						
TV/Screen Min	-159.59	-29.92	**<0.0001**	-171.77	-43.35	**<0.0001**
Total Inactive Min	-69.56	-5.37	**<0.0001**	5.14	0.48	0.64
**WHO QoL BREF**[e]						
QoL Rating	0.30	12.14	**<0.0001**	0.24	9.39	**<0.0001**
Health Rating	0.07	2.99	**0.003**	-0.07	-3.18	**0.002**
Health Satisf.[f]	0.48	15.60	**<0.0001**	0.59	20.98	**<0.0001**
Physical	0.10	5.38	**<0.0001**	-0.05	-3.28	**0.001**
Psychological	0.16	9.37	**<0.0001**	-0.01	-0.64	0.52
Social	-0.05	-2.40	**0.02**	0.01	0.68	0.50
Environmental	-0.08	-4.69	**<0.0001**	0.02	1.52	0.13
**Social Support for Exercise**						
Family Participation	2.17	25.81	**<0.0001**	0.10	1.13	0.26
Family Rewards	0.01	0.98	0.33	-0.08	-5.78	**<0.0001**
Friend Participation	2.42	27.01	**<0.0001**	0.98	11.30	**<0.0001**

[a] BP= Blood Pressure; HR= Heart Rate; BMI= Body Mass Index.

[b] CHAMPS= Community Healthy Activities Model Program for Seniors Questionnaire. PA= Physical Activity.

[c] Min= Minutes.

[d] MVPA= Moderate-to-Vigorous Physical Activity.

[e] WHO QoL BREF= World Health Organization Quality of Life; BREF= Best available technique reference document.

[f] Satisf.= Satisfaction.

Note: For the CHAMPS, WHO Quality of Life, and Social Support for Exercise instruments, higher numbers reflect more positive responses/outcomes. For the Physical Inactivity questionnaire, higher numbers reflect more inactivity/sedentary behaviors.

© 2025 King AC et al. *JAMA Network Open*.

eTable 2: Between-Arm Program Advisor Satisfaction Ratings at 12 Months: Working Alliance Inventory (Bond Subscale) Results

Outcome	Mean (SD)	B (SE)	90% CI	*p* Value
Total Bond subscale				
Bond	5.86 (1.51)	0.58 (0.18)	0.56, 0.60	**0.0001**
Human Advisor Arm	6.16 (2.36)	–	–	–
SMS Advisor Arm	5.57 (0.12)	–	–	–
Individual Bond items				
PAA likes me	–	1.35 (0.30)	1.32, 1.37	**<0.0001**
PAA mutual respect	–	0.61 (0.23)	0.59, 0.63	**0.009**
Confidence in PAA	–	0.55 (0.24)	0.53, 0.57	**0.03**
PAA mutual trust	–	1.15 (0.29)	1.12, 1.18	**<0.0001**

[a]B = Beta Coefficient; CI = Confidence Interval; PAA = Physical Activity Advisor; SD = Standard Deviation; SE = Standard Error

[b]Estimates denote outcomes for the Human Advisor arm with the SMS Advisor arm as the reference group

[c]**Bolded *p* Value** = Significant at ≤ 0.05. The results indicate that, commensurate with other direct comparisons of human vs. computer advisor interventions in the physical activity promotion area,[5,6] human advisor participants rated their advisors more positively than SMS advisor participants.

References

1. King AC, Sallis JF, Frank LD, et al. Aging in neighborhoods differing in walkability and income: Associations with physical activity and obesity in older adults. *Soc Sci Med*. 2011;73:1525-1533. doi:10.1016/j.scoscimed.2011.08.032 http://dx.doi.org/10.1016/j.socscimed.2011.08.032
2. Hart TL, Swartz AM, Cashin SE, Strath SJ. How many days of monitoring predict physical activity and sedentary behaviour in older adults? *Int J Behav Nutr Phys Act*. 2011;8:62-68.
3. Evenson KR, Wen F, Metzger JS, Herring AH. Physical activity and sedentary behavior patterns using accelerometry from a national sample of United States adults. *Int J Behav Nutr Phys Act*. Feb 15 2015;12:20. doi:10.1186/s12966-015-0183-7
4. Evenson KR, Wen F, Herring AH. Associations of Accelerometry-Assessed and Self-Reported Physical Activity and Sedentary Behavior With All-Cause and Cardiovascular Mortality Among US Adults. *Am J Epidemiol*. Nov 1 2016;184(9):621-632. doi:10.1093/aje/kww070
5. King AC, Friedman RM, Marcus BH, et al. Ongoing physical activity advice by humans versus computers: The Community Health Advice by Telephone (CHAT) Trial. *Health Psychol*. 2007;26:718-727.
6. King AC, Campero MI, Sheats JL, et al. Effects of Counseling by Peer Human Advisors vs Computers to Increase Walking in Underserved Populations: The COMPASS Randomized Clinical Trial. *JAMA internal medicine*. Sep 28 2020;180(11):1481-1490, doi:10.1001/jamainternmed.2020.4143

© 2025 King AC et al. *JAMA Network Open*.
