# Supplementary material for: Human Telephone vs Text Message Counseling and Physical Activity Among Midlife and Older Adults: A Randomized Clinical Trial
Source: JAMA Netw Open. 2025 Sep 4;8(9):e2528858. doi: 10.1001/jamanetworkopen.2025.28858 (PMC12411977; doi:10.1001/jamanetworkopen.2025.28858)
Supplement: Supplement 3. — Data Sharing Statement [file jamanetwopen-e2528858-s003.pdf]

Data Sharing Statement

King. Human Telephone vs Text Message Counseling and Physical Activity Among Midlife and Older Adults. *JAMA Netw Open*. Published September 03, 2025.
doi:10.1001/jamanetworkopen.2025.28858

Data
Additional Information: ClinicalTrials.gov Identifier: NCT02385591
Data available: Yes
Data types: Deidentified participant data
How to access data: Data types: Behavioral, psychosocial, clinical Additional Information: We will make the final data collected and described in current publications available in electronic form to researchers who request them as part of the following data approval process: We will request that researchers submit a specific data request in writing to the project principal investigator so that the requested data can be made available while protecting the confidentiality and rights of study participants, avoiding overlap with planned data analyses by the investigator group, and limiting duplication of data analysis activities. How to access data: See above. The email address to request the data is as follows: king@stanford.edu (Abby King, study PI) When available: With publication Supporting Documents: Document types: None Additional Information: Who can access the data: Researchers whose proposed use of the data has been approved by the principal investigator and study investigation team Types of analyses: The types of analyses for which the data will be made available include those that extend the knowledge base in the field and avoid or limit duplication with investigator-planned or conducted analyses. Mechanisms of data availability: Data will be made available with investigator support after approval of a proposal and an accompanying signed data access agreement which follows the stipulations and guidelines of Stanford University School of Medicine.
When available: With publication

Supporting Documents
Document types: None

Additional Information
Who can access the data: Researchers whose proposed use of the data has been approved
Types of analyses: For a specified purpose that has been approved by the investigative team
Mechanisms of data availability: After approval of a proposal and with a signed data access agreement
